# Supplementary material for: Chromothripsis during telomere crisis is independent of NHEJ, and consistent with a replicative origin
Source: Genome Res. 2019 May;29(5):737–49. doi: 10.1101/gr.240705.118 (PMC6499312; doi:10.1101/gr.240705.118)
Supplement: Supplemental Material [file supp_gr.240705.118_Supplemental_file_1.zip › contigs/annotated_contigs/DB57/contig.2.DB57_length_354_mean_cov_21.0480225989.docx]

**DB57_length_354_mean_cov_21.0480225989**

```
CTCGCGGGCTCTCCACTGCGTCGCCTGCTCTTTCCTTAC|TGGTGGGCTCTTCCGGGCGCCGCCTGGCGTAGCGTCTGTGGTGGGCTCT
                                        >chr7:157235131-157235269 + E=1e-62
TCTGGTCGGCGCCTGACGTAGCGTCTGTCGTGGGCTGTTCGGGGCGGGCGCCTGATGTAGCGTCTGTGGTGGACTTTCCGGGTGGTG|A

C|GAAAAGAGTAAAATCCCCTGTTACTGGTTCTAAACAAAGCTTGAGTAAAGACTTCATGTTATATCACTAGGAGTTTCTTGGCTTCAT
>chr7:146036710-146036889 - E=2e-96
TCACATCTACTTTTGTTTATTCATAATTTCCATATAGACCTAAGTTATCTGCCAGTAGCTCTAGTTACATATATTTAAATAGATATTCT

T
```
